# Updated general recommendations in cancer management during the COVID-19 pandemic in the Philippines

**DOI:** 10.3332/ecancer.2020.1128

**Published:** 2020-10-20

**Authors:** Frederic Ivan Ting, Marvin Jonne Mendoza, Danielle Benedict Sacdalan, Honey Sarita Abarquez

**Affiliations:** 1 Riverside Bacolod Cancer Care Center, 6100 Negros Occidental, Philippines; 2 University of the Philippines—Philippine General Hospital, 1000 Manila, Philippines; 3 Davao Doctors Hospital, 8000 Davao del Sur, Philippines; 4 Perpetual Succour Hospital, 6000 Cebu, Philippines

**Keywords:** COVID-19, recommendations, management, cancer patients, Philippines

## Abstract

In May 2020, the Philippine Society of Medical Oncology published its initial recommendations on the treatment of cancer patients during the SARS-Cov-2 pandemic. The objective of this update is to provide answers to the questions pertaining to the diagnostic testing of SARS-CoV-2 for both cancer patients and healthcare professionals caring for cancer patients, as well as the recommended protective measures and practices that may be instituted in healthcare facilities.

## Introduction

Six months into the SARS-CoV-2 pandemic, the Philippines continues to struggle in coping with the increase in cases, especially during the last weeks of July 2020. As the nation went on an enhanced community quarantine in March 2020, the healthcare community learned to cope with the demands of caring for those affected by the virus, adopting and applying effective medical practices as evidence unfolded. To cope with the increasing number of infected individuals, medical facilities were converted into COVID-19 referral centres and Filipinos were advised to stay at home as part of the preventive measures to limit the contagion.

Despite the country’s low COVID-19 mortality rate, SARS-CoV-2 continues to impact all Filipinos, particularly those with medical conditions like cancer, cardiovascular, pulmonary and other comorbid diseases. While real-time reverse transcriptase-polymerase chain reaction (rRT-PCR) diagnostic laboratories have been set up throughout the country to identify those affected by the virus, capability and resources to pay for the tests remain very limited relative to the country’s population. Thus, the preventive measures of hand washing, physical distancing, wearing of masks and face shields remain the most cost-effective measures to save all lives.

Similar to the situation in different parts of the world [1–3], the COVID-19 pandemic has had a significant impact on the management of cancer patients in the Philippines in terms of diagnosis and treatment, considering the increased risk of infections and the negative consequences in patients with cancer [4, 5]. These patients may also be at an increased risk of developing severe events related to COVID-19 compared with the general population [6–8].

The uncertainties brought about by this public health emergency have likewise presented unique challenges to oncologists around the world [9, 10], with one study showing potentially alarming signals of undertreatment [11]. Despite these difficulties, physicians strive to continue to provide compassionate and safe care to cancer patients.

In May 2020, the Philippine Society of Medical Oncology (PSMO) published its initial article on the treatment of cancer patients during the SARS-Cov-2 pandemic, dwelling on the prioritisation of cancer care, ensuring a safe work environment, organising transition of cancer care and maintaining cohesion in a time of isolation [12]. However, the application of these guiding principles in a real-world setting has remained unclear for medical oncologists who are faced with dilemmas on testing for COVID-19 among patients and healthcare workers.

The objective of this article is to provide specific answers to questions pertaining to the diagnostic testing of SARS-CoV-2 for both cancer patients and healthcare professionals caring for cancer patients, as well as the recommended protective measures and practices that may be instituted in healthcare facilities. This article reviews recommendations from the American Society of Clinical Oncology, the European Society of Medical Oncology, and the PSMO’s recommendations on how these practices may be adapted by each medical oncologist providing oncologic care throughout the archipelago.

## Recommendations on COVID-19 testing for cancer patients

Multiple studies carried out around the world have shown that patients with cancer are at a higher risk for severe complications and mortality from SARS-CoV-2 infection [13–19]. In the scientific brief by the World Health Organisation (WHO) on July 11, 2020, the organisation acknowledged the possibility of airborne transmission in addition to the droplet, contact, fomite, faecal–oral, bloodborne, mother-to-child and animal-to-human transmission. Furthermore, the WHO confirmed that SARS-CoV-2-infected persons without symptoms can also infect others [20]. Guided by this assumption and in the dictum of *primum non nocere*, the identification of SARS-CoV-2 infection among cancer patients became an utmost priority.

### Prioritisation of cancer patients who need testing

PSMO acknowledges that protocols regarding prioritisation of patients may vary according to institutional resources and local testing capacity. For general guidance, prioritisation of cancer patients for COVID-19 testing was adopted from previous recommendations by international oncologic societies [21, 22] (Table 1).

### Proper screening of patients

Consistent with our previous recommendations, a triage/screening area manned by personnel with appropriate personal protective equipment (PPE) should be in place in all cancer institutions to ensure the safety of both patients and healthcare workers [12, 23].

Obtaining a good history for signs and symptoms of COVID-19 is paramount. Systematic reviews of observational studies showed that the common symptoms of COVID-19 are fever, cough, fatigue /myalgia, dyspnoea, shortness of breath, chest tightness/pain, anorexia, arthralgia, sputum production, chills, headache and sore throat. Less common symptoms (<10%) include diarrhoea, abdominal pain, dizziness, rhinorrhoea, nausea and vomiting, nasal congestion and haemoptysis. Patients presenting with dyspnoea or shortness of breath were noted to be at a higher risk of developing severe cases of COVID-19 or death [24].

Furthermore, the use of a standardised questionnaire (e.g., Philippine College of Physicians – Philippine Society of Microbiology and Infectious Disease (PCP–PSMID) OPD Patient Screening Form) to screen for symptoms and potential exposure is recommended [25].

### Types of test

1. **rRT-PCR assay**
  - **An rRT-PCR using nasopharyngeal swab specimens should be carried out to confirm the presence of SARS-CoV-2 infection among cancer patients whenever feasible.**
  - The currently recommended test and the ‘gold standard’ to confirm COVID-19 infection is rRT-PCR (real-time reverse transcriptase – polymerase chain reaction) assay. Using this assay, SARS CoV-2 viral RNA can be detected in nasal or pharyngeal samples, sputum, bronchoalveolar lavage fluid and other bodily fluids, including faeces and blood [26].
  - Among the upper respiratory tract specimens, nasopharyngeal and nasal swabs had the highest sensitivity (97% and 95%, respectively) with a specificity of 100% [27].
2. **Rapid tests based on antigen production**
  - It is important to note that the WHO does **not** currently recommend the use of antigen-detecting rapid diagnostic tests for patient care, although research into their performance and potential diagnostic utility is highly encouraged [29].
  - **However, in centres where rRT-PCR diagnostic assay for COVID-19 is not available, the rapid antigen tests can potentially be used as an alternative among symptomatic cancer patients during the first week of illness**. Negative results from an antigen test should be **confirmed** with an RT-PCR test prior to making treatment decisions to prevent the spread of the virus. Oncologists are advised to interpret the results with utmost care and caution, and to always correlate it with clinical and epidemiologic parameters [26].
  - Rapid antigen test detects the presence of viral proteins (antigens) expressed by the COVID-19 virus in a sample from the respiratory tract of a person. The antigen(s) detected are expressed only when the virus is actively replicating; therefore, such tests are best used to identify acute or early infection [26].
  - Although commercially available rapid antigen tests report a sensitivity of 87% (95% CI 52.9–97.8) and a specificity of 100% (95% CI 96.8–100) based on a small study using direct nasal swabs from sequentially enrolled patients compared to a SARS CoV-2 extracted RT-PCR assay, some studies show a very low overall sensitivity rate of 30.2% [28].
3. **Detection of Antibodies to SARS-CoV-2**
  - **The use of IgM/IgG rapid test kit is not recommended in the diagnosis of COVID-19 in cancer patients.**
  - Two clinical trials showed that there is insufficient evidence to support the use of IgM/IgG rapid test kits for the definitive diagnosis of COVID-19. Diagnostic accuracy varies greatly depending on the timing of the test. Furthermore, the test performed very poorly during the early phase of the disease (e.g., <8 days from the onset of symptoms) [30, 31].
  - The PCP-PSMID guidelines also do **not** recommend rapid point-of-care lateral flow immunoassay antibody tests as stand-alone tests for the diagnosis of COVID-19.
  - The WHO also does **not** currently recommend the use of antibody-detecting rapid diagnostic tests for patient care but encourages the continuation of ongoing research to establish its usefulness in disease surveillance [29].

### Timing of the test

- **An rRT-PCR SARS-CoV-2 nasopharyngeal swab test should be conducted for cancer patients presenting with COVID-19 signs and/or symptoms.**
- **An rRT-PCR SARS-CoV-2 nasopharyngeal swab test is recommended for new asymptomatic cancer patients BEFORE receiving their first cycle of immunosuppressive therapy** which includes, but is not limited to, cytotoxic chemotherapy, biologic therapy, immunotherapy, high-dose corticosteroids, extensive radiotherapy or stem cell transplantation. Ideally, the test should be conducted **48–72 hours** prior to the start of the immunosuppressive treatment if patient resources or institutional logistics allow [21, 22]. *If the test is found impractical to do by the attending oncologist because of logistical/financial concerns or the patients’ clinical scenario, please see ‘For Areas where rRT-PCR SARS-CoV-2 Testing is NOT Readily Available’ below.*
- **For asymptomatic cancer patients who are on succeeding cycles of immunosuppressive treatment, the oncologist has the option to screen patients using repeat rRT-PCR SARS-CoV-2 nasopharyngeal swab test or the 14-day COVID-19 symptom-based test.** A systematic review showed that among asymptomatic individuals with possible exposure to COVID-19, the sensitivity of detecting active COVID19 infection with a 14-day symptom-based test is 92.8% and specificity is 98.3% [32]. It is important to take note that the latter test was conducted in healthy individuals. However, in resource-limited settings and with the limited studies available for cancer patients, the Society recommends that the oncologists assess the individual patient’s clinical scenario and apply the available evidence judiciously with consideration to patient resources and institutional logistics.
- It is important to note that the sensitivity and specificity of a test vary with time, from symptom onset due to changes over time in the viral load [33].
- Using rRT-PCR, the probability of detecting SARS-CoV-2 varies based on time from the exposure, being as low as 0% in the immediate days following exposure, 33% one day before symptom onset, 62% on the day of symptom onset and peaking at 80% on day 3 of symptoms [34].

### For areas where rRT-PCR SARS-CoV-2 testing is NOT readily available

- **Symptomatic Cancer Patients**
  - In settings where the rRT-PCR SARS-CoV-2 test is unavailable, or the logistical scenario makes its use difficult, **the recommendations of PCP-PSMID that rapid antigen tests can potentially be used as an alternative among symptomatic cancer patients during the first week of illness may be applied.** Negative results from an antigen test should be **confirmed** with an rRT-PCR test prior to making treatment decisions to prevent the spread of the virus. Oncologists are advised to always correlate with clinical and epidemiologic parameters [26].
- **Asymptomatic Cancer Patients who will start or are on active treatment**
  - One strategy to ensure patients arrive for their immunosuppressive cancer treatment without subclinical SARS-CoV-2 infection is to observe a period of **strict self-quarantine for 14 days**, if logistically possible [25].
  - This may be an alternative for screening asymptomatic cancer patients in resource-limited settings since any prior exposure would have sufficient time to enter the symptomatic phase. However, with the data on asymptomatic COVID-19 cases rising, the **oncologist should discuss the best available evidence (including the risks of chemo if there is undetected COVID-19 infection) to patients prior to making an informed decision.** Furthermore, not all patients will have the means to self-quarantine and this strategy may add to the already apparent social and economic burden of cancer treatment.

### Patients who are SYMPTOMATIC with POSITIVE rRT-PCR results

- **Cancer patients with confirmed COVID-19 infection should be referred to an Infectious Disease specialist and managed according to the latest PCP-PSMID-PCCP Interim Guideline**s [26].
- **Immunosuppressive treatment for these patients should be delayed for 10–14 days after symptom onset and/or cleared by infectious disease expert based on clinical parameters** [21, 22]. Furthermore, the patient should be symptom-free and improving for a minimum of 72 hours prior to treatment.
- **There is no gold standard ‘test of cure’ for COVID-19** [26]. Based on the available data [29], a test-based or symptom-based strategy may be used. For persons who are immunocompromised, including cancer patients on chemotherapy, a test-based strategy could be done, preferably in consultation with infectious disease experts. For patients who are already asymptomatic but still have persistently positive rRT-PCR results, guidance from an infectious disease expert is recommended.
- Currently, the need for documenting two consecutive rRT-PCRs is being questioned in light of recent evidence showing that although viral shedding can last several weeks, the virus is likely to be non-viable by day 8 [26]. The US-CDC acknowledges that detecting viral RNA via rRT-PCR does not necessarily mean that an infectious virus is present [41].
- If a confirmed case was noted to have entered the clinic or hospital grounds, the area where the patient was noted to have stayed should be disinfected accordingly and contact tracing protocols should be set in place according to the PSMID Unified COVID-19 Algorithms [42].

### Patients who are SYMPTOMATIC but with NEGATIVE rRT-PCR results

- **REPEAT TESTING** for patients with an initial negative COVID-19 test result should be carried out if there is a **high index of suspicion** for COVID-19 infection, despite an initial negative test result [26].

### Patients who are ASYMPTOMATIC but with POSITIVE rRT-PCR results

- **Immunosuppressive treatment for these patients should be delayed until a repeat rRT-PCR result shows a negative result and/or cleared by an infectious disease specialist** [43].

### Patients who are ASYMPTOMATIC and with NEGATIVE rRT-PCR results

- Cancer patients who are asymptomatic with a negative test result may proceed with the planned immunosuppressive treatment after being clinically assessed by his/her oncologist.

### Ancillary tests

- **Symptomatic patients**
  - The following ancillary tests are recommended when COVID-19 is suspected to guide management if deemed imperative by the attending physician: complete blood count; metabolic panel: creatinine, liver function tests, sodium, potassium, magnesium, calcium and albumin; inflammatory markers: lactate dehydrogenase, ferritin, C-reactive protein and procalcitonin; prothrombin and D-Dimer; arterial blood gas measurement; blood cultures if concomitant bacterial infection is suspected; respiratory tract specimen for influenza testing; sputum, endotracheal aspirate, or bronchoalveolar lavage fluid culture and sensitivity; chest X-ray; high-resolution chest CT scan plain and ECG [26].
- **Asymptomatic cancer patients**
  - Although **chest X-ray **is used as an initial diagnostic tool in some institutions due to its availability and the delay in results of RT-PCR, **its universal use as a screening test is not recommended because of its low sensitivity (69% versus 91% for rRT-PCR), especially early in the disease; moreover, abnormalities, if detected, are non-specific** [36].
  - A systematic review shows the following chest X-ray findings among laboratory-confirmed COVID-19 patients [37]:
    - Chest X-ray typically shows ground-glass opacities and consolidation in the lung periphery.
    - Initial imaging results may be normal early in the course of illness and in mildly symptomatic patients.
    - Abnormalities are not specific and overlap with other infections and coronavirus types of pneumonia.
  - ***The universal use of Chest CT scans as a screening tool (for asymptomatic patients) is not recommended.***
  - The characteristic chest computed tomographic imaging abnormalities for COVID-19 are diffuse, peripheral ground-glass opacities [39]. Ground-glass opacities have ill-defined margins, air bronchograms, smooth or irregular interlobular or septal thickening, and thickening of the adjacent pleura. Early in the disease, chest computed tomographic imaging findings in approximately 15% of individuals and chest radiograph findings in approximately 40% of individuals can be normal [40]. The rapid evolution of abnormalities can occur in the first 2 weeks after symptom onset, after which they subside gradually [39, 41].

## Recommendations for healthcare workers handling cancer patients

As front liners taking care of immunocompromised patients in the COVID-19 response, healthcare workers (HCWs) are continuously at risk of acquiring the infection. HCWs could get SARS-CoV-2 at work through direct or indirect contact with infected patients or other healthcare workers, or as a result of ongoing community transmission [44]. A study in the United Kingdom and the United States estimated that frontline healthcare workers had a 3.4-fold higher risk than people living in the general community for reporting a positive test, adjusting for the likelihood of receiving a test [45]. Approximately 3.8%–10% of the confirmed COVID-19 positive cases in China, Italy and Spain were healthcare workers [46, 47].

In June 2020, a hospital-wide healthcare worker surveillance testing programme conducted at the Philippine General Hospital revealed a relatively low COVID-19 rRT-PCR positivity rate among healthcare workers.

Of 4871 HCWs, 99 (2%) tested positive. Twenty-six of 1,794 (1.4%) assigned to the COVID wards and intensive care unit (ICU) tested positive, while 16 of 893 (1.8%) assigned to the non-COVID areas were positive. Ten (1.16%) of 858 HCWs with essential but non-clinical work were positive. Of the 439 HCWs with symptoms, 21 (4.7%) tested positive. Of the 4,432 HCWs without symptoms, 78 (1.4%) tested positive for COVID-19 [48]. This shows that with strict adherence to institutional safety protocols, transmission rates of COVID-19 among healthcare workers could be minimised [49].

Measures to prevent transmission in specialised facilities, such as cancer centres, are paramount and an immediate priority in order to: 1) safeguard and protect immunocompromised patients and the healthcare staff; 2) slow the demand for specialised healthcare, such as use of mechanical ventilators and ICU beds; and 3) minimise the export of cases to other healthcare facilities and the general community. As such, the following recommendations were made to guide HCWs handling cancer patients on immunosuppressive therapy.

### General recommendations

1. Workplace risk assessments should be conducted routinely and the appropriate measures to continuously minimise exposure to and transmission of the virus should be implemented. This includes, but is not limited to, the following:
  - Minimise social gathering (i.e., avoid eating in a communal area and relaxing in lounge or common areas without face masks, cancel face-to-face conferences, among others).
  - Adhere to the minimum standard PPE recommendations and transmission-based precautions set by the respective institutions (See No. 7).
  - Restructure the clinic or facility, as applicable, in accordance with the hospital infection control unit and/or safety and health committee recommendations.
  - Observe proper respiratory/coughing etiquette and disposal of used hygiene materials.
  - Decontaminate the workplace regularly following hospital and local recommendations.
  - Provide alternative ways of doing operations (i.e., online platforms for conferences and meetings, telemedicine for patients, as appropriate) [21, 22].
  - Limit the number of visitors or companions during admissions or clinic visits.
2. All HCWs should be updated on the epidemiology of COVID-19 in their respective institutions and localities, including the known risk factors for infection, the presenting clinical signs and symptoms and the procedures for reporting, testing, isolating or transferring co-workers who are probable, suspected or confirmed COVID-19 cases. Emerging evidence and evolving guidance should be taken into account.
3. The staff should be provided with the necessary education, training and assessment on the following [21, 22]:
  - Infection control, proper selection and use and disposal of personal protective equipment and proper use of respirators.
  - Proper use and cleaning of equipment, clinic rooms, chemotherapy infusion facilities, among others.
  - Proper disposal of potentially infectious wastes.
4. Institutions should have established COVID-19 consultation hotlines and health service system to triage, examine and provide immediate dispositions (i.e., testing, contact tracing, isolation and quarantine period and return to work policies) on symptomatic and/or infected HCWs [46]. An organised communication protocol or algorithm for notification of the appropriate public health authorities should be in place.
5. As much as possible, HCWs working on cancer care facilities should not be mixed with HCWs providing care for COVID-19 patients to avoid cross-contamination by undocumented, asymptomatic medical personnel [23, 50]. All physical contact between staff should be minimised and observance of physical distancing should be maintained whenever possible [21, 22].
6. Identify the number of staff essential for facility operations and patient care and treatment [6]. As much as possible, minimal staff members should be involved in direct care of cancer patients during their treatment days [46].
7. All HCWs handling cancer patients should always adhere to the minimum standard and transmission-based precautions for COVID-19, which include [51]:
  - Use of face shield or goggles [52].Use of facemasks.
  - Use of N95 masks or higher-level respirator based on anticipated exposures and suspected or confirmed diagnoses [53]. A recent systematic review and meta-analysis [51] estimated that respirators may provide a stronger protective effect compared to medical masks.
  - Observance of physical distancing of 1 meter or more.
  - Strict hand hygiene after every patient encounter and/or contact with potentially contaminated surfaces.
8. Periodic HCW risk assessment should be conducted to allow for early identification, immediate work restriction and monitoring of HCWs either at high risk* of exposure or who were exposed to confirmed or suspected COVID-19 cases.
  - Self-monitoring of fever, respiratory symptoms and other symptoms related to COVID-19.
  - Recommendations on testing (*see below*).
  - Low-risk HCWs who report potential exposure and are asymptomatic are NOT excluded from work [21, 22].
  *High-risk exposure is defined as:
  - Close contact with a confirmed COVID-19 patient in the community.
  - Provision of direct patient care for a patient with COVID-19 without the adequate use or provision of proper PPE.
  - Contact with infectious secretions from a confirmed COVID-19 patient or contaminated patient care environment without the adequate use or provision of proper PPE.
9. If a particular HCW is deemed to have an increased risk for severe complications or death when infected with COVID-19, they should be reassigned away from the *high-risk areas* (COVID-19 wards) [54]. These may include:
  - HCWs with diabetes.
  - HCWs with predisposed cardiac condition.
  - HCWs with asthma.
  - HCWs who are obese and with obstructive sleep apnoea.
10. All HCWs caring for cancer patients with symptoms compatible with COVID-19 should be immediately relieved from their duties and isolated while symptomatic, and should be prioritised for testing in order to be able to return to work as soon as possible, with clearance from the hospital infection control unit and/or safety and health committee.

### Recommendations on rRT-PCR testing

While molecular testing with rRT-PCR is widely available in the National Capital Region (NCR) and Metro Cebu, testing resource constraints continue in the rest of the archipelago. For this main reason, it is prudent to continue to use an evidence-based approach when making COVID-19 testing priorities.

Testing of HCWs working in cancer facilities should be considered in the following situations [55]:

- HCWs with signs or symptoms consistent with COVID-19.
- Asymptomatic HCWs with known or suspected exposure to SARS-CoV-2.
- HCWs who have been diagnosed with COVID-19 to determine when they are no longer infectious, or prior to returning to work, as deemed necessary by the hospital infection control unit and/or safety and health committee.

1. When testing is feasible and available, HCWs should be considered a priority for evaluation [54]. Facilities should consider COVID-19 testing for all HCWs at the beginning of a cycle of consecutive workdays [21, 22].
2. In case of limited testing capacity, symptomatic HCWs should be prioritised for testing over low-risk groups [56].
3. If no testing is available or testing capacity is limited, for the purposes of returning to work, symptom-based strategy must be employed. HCWs should be excluded from work until:
  - At least 10 days since symptoms first appeared, AND
  - At least 3 days with no fever without anti-pyretics AND symptoms have improved [46].
  True asymptomatic infection is believed to be uncommon. The average time from exposure to symptoms onset is 5 days, and up to 62% of transmission may occur prior to the onset of symptoms. Although studies have described rates of asymptomatic infection, ranging from 4% to 32%, it is unclear whether these reports represent truly asymptomatic infection by individuals who never develop symptoms, transmission by individuals with very mild symptoms or transmission by individuals who are asymptomatic at the time of transmission but subsequently develop symptoms [57].
4. For asymptomatic HCWs with COVID-19 infection, they may be cleared to return to work using the following:
  - Time-based strategy. Exclude from work until:
    - 10 days have passed since the date of their first positive COVID-19 diagnostic test, assuming they have not subsequently developed symptoms since their positive test. If they develop symptoms, then the symptom-based or test-based strategy should be used. Note that because symptoms cannot be used to gauge where these individuals are in the course of illness, it is possible that the duration of viral shedding could be longer or shorter than 10 days after their positive test.
  - Test-based strategy. Exclude from work until:
    - Negative results for SARS-CoV-2 from at least two consecutive respiratory specimens collected ≥24 hours apart (total of two negative specimens). Note, because of the absence of symptoms, it is not possible to gauge where these individuals are in the course of their illness. There have been reports of prolonged detection of RNA without direct correlation to viral culture
  Protecting the health of HCWs is paramount. The well-being of HCWs can be promoted by the provision of adequate PPE, as well as by ensuring that infected colleagues are promptly tested and isolated. The scale of asymptomatic HCWs positive with COVID 19 is not fully understood, nor is the full potential for asymptomatic and pre-symptomatic HCWs to transmit infection to patients who do not have COVID-19, other HCWs or the public. However, given that asymptomatic transmission has been documented, utmost caution is urged [58].
5. Timing of RT-PCR testing
  - Sensitivity of testing varies with the timing of testing relative to exposure [34, 57]
    - Day 1 of exposure: 0%
    - Day 4 of exposure: 33%
    - Day 5 of exposure, Day 1 of symptoms: 62%
    - Day 8 of exposure, Day 3 of symptoms: 80%
    - Day 21 of exposure, likely no more symptoms: 34%
  - Care must be taken in interpreting rRT-PCR tests for SARS-CoV-2 infection—particularly early in the course of infection—when using these results as a basis for removing precautions intended to prevent onward transmission. If clinical suspicion is high, infection should not be ruled out on the basis of rRT-PCR alone, and the clinical and epidemiologic situation should be carefully considered [34].
6. Should there be a case of confirmed COVID-19 among the HCWs tested, the institution should have an established communication protocol or algorithm, as mentioned above.

### Contingency plans in cases of HCWs’ massive infection and staff shortage

- At times of surge or massive infection in the cancer care facility, the recommendations will have to be revisited to allow for adequate workforce support to continue providing optimal, appropriate and compassionate cancer care, while still prioritising the safety of both patients and healthcare staff [46].
- Institutions should consider splitting healthcare teams to ensure continuity of cancer care during the COVID-19 pandemic [59].
- Should cancer facilities have staffing shortages, the institution may allow asymptomatic HCWs with higher risk exposures to continue to work during their 14-day post-exposure period. If testing is available, carrying out testing during the 14-day post-exposure period can be considered in order to quickly identify pre-symptomatic or asymptomatic carriers. For HCWs with lower risk exposures, symptom screening and source control measures while at work should be in place [55].
- Appropriate information and training to HCWs recruited for the temporary replacement of the quarantined workforce should be provided.
- An adaptable framework for possible transfer of clinical care and/or continuity of management should be in place to allow for quick referral and coordination of affected patients.
- Cancer centre leaders should be attentive to HCWs who may experience increased stress, anxiety, burn-out or depression due to the COVID-19 pandemic. Psychological support and stress management resources should be made available to all HCWs to maintain physical and emotional well-being and to provide effective coping strategies [21, 22].

## Conclusion

This review and recommendations were put together by PSMO for Filipino medical oncologists to provide safe and effective cancer care during the COVID-19 pandemic. As new data emerge, the PSMO fervently supports the dictum of safety and well-being for all cancer patients and healthcare workers.

## Cancer-specific guidelines

Site-specific cancer care guidelines during the COVID-19 pandemic will also be available at www.psmo.org.ph.

## Funding declaration

This work did not receive any funding.

## Conflicts of interest

The authors have no conflicts of interest to declare.

## Disclaimer

Data presented here are based on the best current evidence as of this writing. However, information about COVID-19 is rapidly evolving, and new evidence may have emerged by the time this article is published. Information contained in this article does not substitute for the independent professional judgment of the medical oncologist or other physicians in the context of treating an individual patient. This document is for informational purposes only and does not constitute medical or legal advice.

## Figures and Tables

**Table 1.** Prioritisation of cancer patients who need COVID-19 testing.

1st Priority	Hospitalised cancer patients with COVID-19 symptoms including, but not limited to, cough, shortness of breath, fever, chills, myalgias, sore throat, new loss of taste or smell, loose bowel movement or other flu-like symptoms.
2nd Priority	Cancer patients seen in the outpatient clinics with COVID-19 symptoms
3rd Priority	Cancer patients who are asymptomatic but are scheduled to receive active therapy that can suppress the immune system (e.g., cytotoxic chemotherapy, biologic therapy, immunotherapy, high-dose corticosteroids, extensive radiotherapy or stem cell transplantation). Cancer patients who are asymptomatic, but have exposure to a confirmed case of COVID-19
